# LaCrO_3_–CeO_2_-Based Nanocomposite Electrodes for Efficient Symmetrical Solid Oxide Fuel Cells

**DOI:** 10.1021/acsaem.1c04116

**Published:** 2022-04-05

**Authors:** Javier Zamudio-García, José M. Porras-Vázquez, Enrique R. Losilla, David Marrero-López

**Affiliations:** † Departamento de Química Inorgánica, Universidad de Málaga, Campus de Teatinos s/n, 29071 Málaga, Spain; ‡ Departamento de Física Aplicada I, Universidad de Málaga, Campus de Teatinos s/n, 29071 Málaga, Spain

**Keywords:** LaCrO_3_, CeO_2_, nanocomposite electrode, symmetrical solid oxide fuel cell, spray pyrolysis

## Abstract

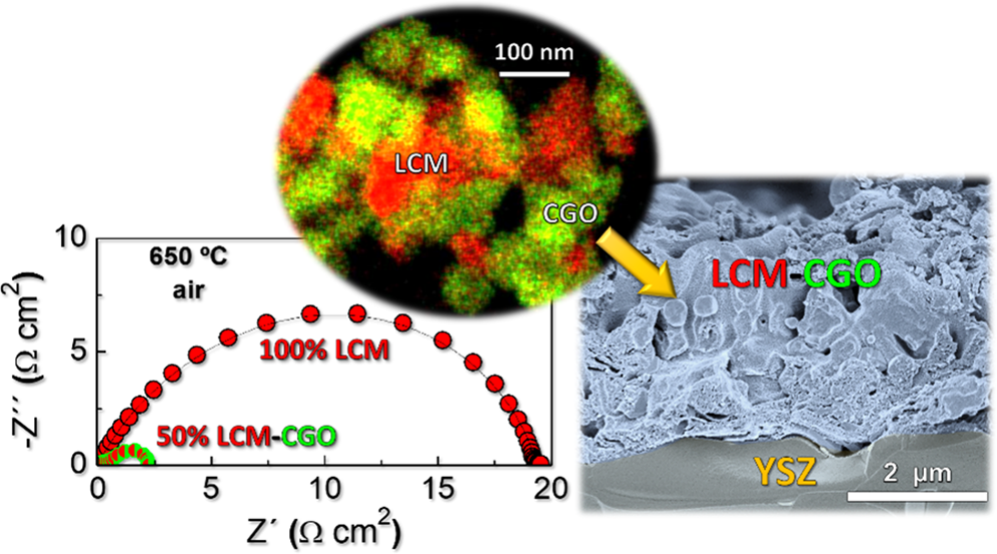

La_0.98_Cr_0.75_Mn_0.25_O_3−δ_–Ce_0.9_Gd_0.1_O_1.95_ (LCM-CGO) nanocomposite layers with different LCM contents, between 40 and 60 wt %, are prepared in a single step by a spray-pyrolysis deposition method and evaluated as both air and fuel electrodes for solid oxide fuel cells (SOFCs). The formation of fluorite (CGO) and perovskite (LCM) phases in the nanocomposite electrode is confirmed by different structural and microstructural techniques. The intimate mixture of LCM and CGO phases inhibits the grain growth, retaining the nanoscale microstructure even after annealing at 1000 °C with a grain size lower than 50 nm for LCM-CGO compared to 200 nm for pure LCM. The synergetic effect of nanosized LCM and CGO by combining their high electronic and ionic conductivity, respectively, leads to efficient and durable symmetrical electrodes. The best electrochemical properties are found for 50 wt % LCM-CGO, showing polarization resistance values of 0.29 and 0.09 Ω cm^2^ at 750 °C in air and H_2_, respectively, compared to 2.05 and 1.9 Ω cm^2^ for a screen-printed electrode with the same composition. This outstanding performance is mainly ascribed to the nanoscale electrode microstructure formed directly on the electrolyte at a relatively low temperature. These results reveal that the combination of different immiscible phases with different crystal structures and electrochemical properties could be a promising strategy to design highly efficient and durable air and fuel electrodes for SOFCs.

## Introduction

The high demand for electrical energy and the need for the protection of the natural environment make the development of an effective infrastructure to produce energy from renewable sources, such as solar and wind, necessary. However, the electrical production from these sources is discontinuous and intermittent to meet the immediate demand;^1,2^ consequently, alternative methods for electrical production and storage are required. In this context, solid oxide cells (SOCs) are of great interest for electrical generation as they can convert the chemical energy of a wide variety of fuels, i.e., H_2_ and hydrocarbons, into electrical energy.^3^ Such devices can also operate reversibly in electrolysis mode to convert the excess of electricity generated by renewable sources into hydrogen, offering numerous advantages compared to rechargeable batteries, such as higher energy density, longer duration, and more flexibility.^4^

A conventional SOC is composed of a dense ceramic ion conductor electrolyte that separates two porous ceramic electrodes with different compositions, typically a mixed ionic–electronic conductor for the air electrode and a Ni-cermet for the fuel electrode.^5^ However, Ni-based anodes present mechanical stability issues upon redox cycling as well as carbon deposition and sulfur poisoning when hydrocarbon fuels are employed.^6^ An alternative cell configuration, known as symmetrical solid oxide cells (SSOCs), where the same electrode material is used as both air and fuel electrodes, has gained great attention in the last few years because of its simpler fabrication process and improved chemical and thermomechanical stability to operate in fuel cell and electrolysis modes.^7^ Nevertheless, the major challenge for the development of SSOCs is to find a suitable electrode with high electrocatalytic activity and adequate long-term stability in both oxidizing and reducing environments.^8,9^

Different symmetrical electrodes have been reported in the literature, including doped LaCrO_3_, SrFeO_3_, PrBa(Fe,Mn)_2_O_5+δ_, and La_0.4_Sr_1.6_MnO_4+δ_.^10−13^ Among them, doped LaCrO_3_ is one of the most widely studied due to its high redox stability, resistance to sulfur poisoning, and carbon coking, as well as chemical compatibility with the traditional Zr_0.84_Y_0.16_O_1.92_ (YSZ) electrolyte. Different approaches have been developed to improve the electrocatalytic properties of LaCrO_3_-based electrodes, including doping in the A- or B-site of the perovskite (A = Ce, Bi and B = Mn, Fe, Ti),^14−16^ and the introduction of highly active catalytic elements, such as Ni and Ru, which are exsolved on the electrode surface upon exposure to reducing atmospheres.^17,18^

Another alternative strategy is the preparation of nanostructured electrodes by infiltration, spray-pyrolysis, and physical deposition methods to increase the triple phase boundary (TPB), where the electrochemical reactions take place.^19^ However, nanostructured electrodes suffer from low thermal stability due to grain growth and coarsening, limiting their application to the low temperature range (400–650 °C).^20^ Hence, the main challenge is to find nanostructured electrodes with sufficient performance and durability for application in the intermediate temperature range (600–800 °C). This can be achieved using self-assembled nanocomposite electrodes consisting of a homogeneous and intimate mixture of two different materials. It has been found that a nanoscale contact between multiple phases inhibits the grain growth and leads to a large concentration of active sites for the electrochemical reactions. Moreover, the strong interphase interaction limits the thermal expansion, thus improving the mechanical properties.^21^ In this context, highly efficient air electrodes have been obtained when mixed ionic–electronic conductors with good electrochemical properties are combined with an oxide-ion conductor, such as doped ceria and zirconia.^22^

In particular, spray pyrolysis has been used to prepare different nanocomposite powder cathodes by a cosynthesis process, such as La_0.6_Sr_0.4_MnO_3−δ_-Zr_0.84_Y_0.16_O_1.92_ (LSM-YSZ)^23^ and Sm_0.5_Sr_0.5_CoO_3−δ_–Ce_0.8_Sm_0.2_O_1.9_ (SSC-SDC).^24^ These nanocomposite cathodes showed an outstanding electrochemical activity when compared to those obtained by physically mixed powders. For instance, Shimada et al. reported a power density over 3 W cm^–2^ at 750 °C for a screen-printed SSC-SDC nanocomposite.^24^ Several nanocomposite cathodes have also been deposited directly by spray pyrolysis on the electrolyte, simplifying the fabrication process and improving the adherence and integrity to the substrate when compared to ink-based depositions.^19,25,26^ This strategy has also been used to obtain Ni-YSZ anodes^27^ but, to the best of our knowledge, there are no studies on mixed oxide nanocomposites for fuel electrodes.

Based on the idea that the combination of materials with different conducting properties can be a promising approach to design new durable and efficient symmetrical electrodes, we have prepared, for the first time, La_0.98_Cr_0.75_Mn_0.25_O_3−δ_–Ce_0.9_Gd_0.1_O_1.95_ (LCM-CGO) nanocomposites with different weight fractions of LCM by spray-pyrolysis deposition. Mn-doped LaCrO_3_ was selected in this study due to its high redox stability and improved performance for fuel oxidation,^28^ while CGO is chemically compatible with LaCrO_3_-based electrodes and exhibits high ionic conductivity. Moreover, the perovskite is A-site-deficient and alkaline-earth-free to prevent possible superficial phase segregation. The nanocomposite electrodes were studied by a wide range of structural, microstructural, and electrochemical techniques to evaluate their potential use as both air and fuel electrodes in solid oxide cells.

## Experimental Section

### Materials Preparation

The electrodes with nominal composition (La_0.98_Cr_0.75_Mn_0.25_O_3−δ_–Ce_0.9_Gd_0.1_O_1.95_, LCM-CGO) and different LCM contents (40, 50, 60, and 100 wt % or 42, 52, 62, and 100 vol %) were prepared in a single step by spray-pyrolysis deposition from aqueous precursor solutions containing stoichiometric quantities of La(NO_3_)_3_·6H_2_O, Cr(NO_3_)_3_·9H_2_O, Mn(NO_3_)_2_·6H_2_O, Ce(NO_3_)_3_·6H_2_O, and Gd(NO_3_)_3_·6H_2_O (Sigma-Aldrich, purity above 99%). The metal concentration of the final solution was 0.02 mol L^–1^ in Milli-Q water for all compositions. Ethylenediaminetetraacetic acid (EDTA) with a concentration of 0.01 mol L^–1^ was added as a chelating agent to prevent precipitate formation. For simplicity, the composition of the different electrodes will be hereafter denoted as *x*LCM, where *x* represents the wt % of LCM. The precursor solutions were sprayed with a flow rate of 20 mL min^–1^, atomized in a spray nozzle, and deposited on different substrate types previously heated at 300 °C. The optimum deposition time and nozzle–substrate distance were 1 h and 25 cm, respectively. After deposition, the layers were calcined in a furnace at 800 °C for 1 h in air with a heating/cooling rate of 2 °C min^–1^ to achieve crystallization.

The nanocomposite layers were first deposited on amorphous quartz wafers of 2 × 4 cm^2^ for better structural characterization and phase quantification by the Rietveld method. The electrochemical characterization in symmetrical cells was performed in Zr_0.84_Y_0.16_O_1.92_ (YSZ) and La_0.9_Sr_0.1_Ga_0.8_Mg_0.2_O_3−δ_ (LSGM) electrolytes. YSZ pellets of 8 mm diameter were prepared from commercial powders supplied by Tosoh, while LSGM powders were synthesized by the freeze-drying (FD) precursor method as described elsewhere.^29^ These powders were compacted into disks of 10 and 1 mm diameter and thickness, respectively, and sintered at 1400 °C for 4 h to reach a relative density greater than 98%.

For comparison purposes, LCM powders were also prepared from the freeze-dried precursor method as described in detail elsewhere.^30^ LCM and Ce_0.9_Gd_0.1_O_1.95_ (CGO, Rhodia) powders (1:1 wt %) were physically mixed by ball-milling with an organic polymeric vehicle (Decoflux). The resulting ink was screen-printed onto the YSZ pellets and sintered at 1100 °C for 1 h to achieve good adhesion to the electrolyte.

### Characterization

The phase formation and crystal structure were analyzed by X-ray powder diffraction (XRD) with an Empyrean PANalytical diffractometer (CuK_α1,2_ radiation). The patterns were analyzed with the X’Pert HighScore and GSAS programs for phase identification and structure analysis, respectively.^31,32^ The redox stability of the nanostructured electrodes was also investigated after successive annealing cycles in 5% H_2_–Ar and air atmospheres.

The morphology was studied by scanning electron microscopy (Helios Nanolab 650, FEI-SEM) and high-angle annular dark-field scanning transmission electron microscopy (HAADF-STEM) in a Talos F200X (FEI-TEM). The grain size distribution was determined by the linear intercept method using the Estereologia software.^33^

Electrochemical impedance spectroscopy was used to determine the electrode polarization resistance with a Solartron 1260 frequency response analyzer in a two-electrode configuration at an open-circuit voltage. The spectra were collected in the frequency range of 0.01–10^6^ Hz with an AC perturbation of 50 mV in air, 5% H_2_–Ar, and wet 100% H_2_ (3 vol % H_2_O) atmospheres, as well as a function of the oxygen partial pressure to differentiate the different rate-limiting steps involved in the oxygen reduction reactions. The effect of a direct current (DC) bias on the electrode performance was analyzed using a three-probe configuration with a circular working electrode of 0.20 cm^2^ and a Pt ring reference electrode surrounding the working electrode.^34^ The impedance spectra were collected with a Zahner XC potentiostat/galvanostat/impedance analyzer. The applied voltage was varied between 0 and 0.4 V in cathodic and anodic polarizations in both air and wet hydrogen atmospheres to evaluate the potential application of the electrodes in fuel cell and electrolyzer modes. The spectra under dc bias were collected over time until no change was observed. The impedance data were processed using equivalent circuit models with the ZView software and distribution of relaxation times (DRT) with DRTtools.^35,36^

To evaluate the efficiency of the LCM-CGO composites in real operating conditions, the 50LCM electrode was deposited symmetrically by spray pyrolysis on a La_0.9_Sr_0.1_Ga_0.8_Mg_0.2_O_3−δ_ (LSGM) electrolyte at 300 °C over an area of 0.20 cm^2^, followed by calcination at 800 °C. A LSGM-supported cell was chosen instead of YSZ due to its higher ionic conductivity, which is crucial to reduce the ohmic losses of the cell. The electrolyte with a thickness and diameter of 300 μm and 13 mm, respectively, was prepared from freeze-dried precursors using the methodology described in a previous report.^30^ The single cell was sealed on an alumina tube support with a glass-ceramic sealant (Ceramabond 668, Aremco). Current–voltage and impedance spectra were collected with a Zahner XC between 650 and 800 °C using static air as oxidant and humidified H_2_ fuel (3% H_2_O), which was passed through a bubbler at 20 °C. The H_2_ flow rate was set at 20 mL·min^–1^ and current–voltage (*I*–*V*) curves were obtained at a scan rate of 10 mV s^–1^.

## Results and Discussion

### Structure of the Electrodes

**Figure 1:**
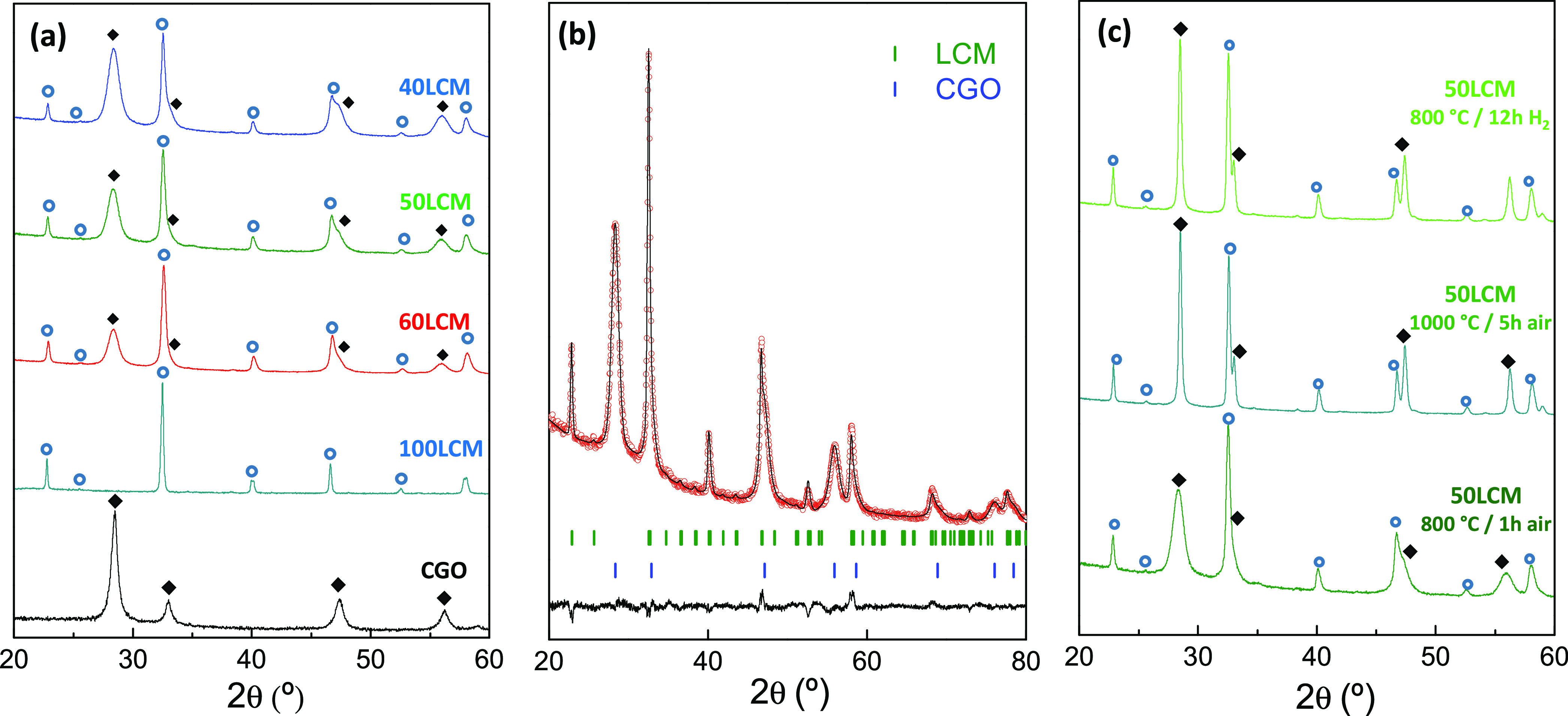
(a) XRD patterns of LCM-CGO nanocomposite electrodes deposited onto quartz substrates and calcined in air at 800 °C for 1 h. Rietveld plots of (b) 50LCM and (c) 50LCM after annealing at different temperatures in air and H_2_.

XRD patterns of the nanocomposite electrodes with different LCM contents and deposited on amorphous quartz substrates are shown in Figure 1a. The pattern of a CGO layer prepared by spray pyrolysis under the same synthetic conditions is also included for direct comparison. The CGO layer crystallizes with a cubic fluorite-type structure in the space group (s.g. *Fm*3̅*m*), while the diffraction peaks of 100LCM matched well with the theoretical pattern of an orthorhombic perovskite-type structure (s.g. *Pbnm*). The patterns of the LCM-CGO nanocomposites show the diffraction peaks of two crystalline phases that are unequivocally assigned to a fluorite and a perovskite-type compound. It is important to remark that additional peaks attributed to secondary phases are not detected.

The XRD patterns were analyzed by the Rietveld method to obtain further insights on the composition and crystal structure of the nanocomposite electrodes. The usual Rietveld parameters were refined during the analysis, i.e., scale factor, zero shift, background, peak-shape asymmetry, and preferential orientation. The cation site occupation factors were fixed and not refined. A representative Rietveld fitting for 50LCM is displayed in Figure 1b and the most relevant structural parameters obtained for all compositions are given in Table 1. Notice that the agreement factor *R*_wp_ varies between 1.9 and 7.1%, thus confirming the accuracy of the fitting.

**Table 1:** Structural and Microstructural Parameters of LCM-CGO Composite Electrodes with Different LCM Contents Deposited on Amorphous Quartz Substrates and YSZ and LSGM Pellets^a^

			volume (Å^3^)					
composition	*T* (°C)	substrate	LCM	CGO	LCM (wt %)	*R*_wp_ (%)	*d*_CGO_ (nm)	*d*_LCM_ (nm)
100LCM	800	FD	235.121(3)		100	3.5		85
100LCM	1000	FD	234.801(4)		100	2.3		205
100LCM (H_2_)	800	FD	235.732(3)		100	3.8		80
100LCM	800	quartz	235.050(2)		100	7.1		59
60LCM	800	quartz	234.934(2)	162.383(2)	58(5)	4.1	10.3	28
40LCM	800	quartz	235.616(2)	161.691(2)	37(4)	4.6	9.5	32
50LCM	800	quartz	235.171(3)	162.017(2)	48(6)	5.6	10.1	31
50LCM	1000	quartz	234.389(2)	159.247(2)	54(4)	4.5	37	41
50LCM (H_2_)	800	quartz	234.843(1)	159.428(2)	52(3)	4.7	36	41
CGO	800	quartz		159.202(4)		2.8	16.0	
50LCM	800	YSZ	235.028(2)	160.953(2)	51(2)	1.9	6.5	47
50LCM	800	LSGM	234.971(5)	161.03(4)	54(2)	3.9	7.2	45

a The data of a conventional powder electrode prepared by the freeze-drying precursor (FD) method is also included for comparison purpose. The particles of LCM and CGO are estimated by Scherrer′s equation.

Regarding the unit cell volumes, it takes a value of 235.050(2) Å^3^ for 100LCM at 800 °C, comparable to that obtained for the corresponding polycrystalline powders from freeze-dried precursors (235.121(3) Å^3^). However, the unit cell volume for LCM in the nanocomposite electrodes decreases slightly with the addition of CGO from 235.616(2) Å^3^ for 40LCM to 234.934(2) Å^3^ for 60LCM. A similar trend is observed for the CGO component: the unit cell volume increases from 159.202(4) Å^3^ for the blank CGO to 162.383(2) Å^3^ for 60LCM, suggesting that a minor cation interdiffusion occurs between CGO and LCM particles during the cosynthesis process. Since the ionic radius of La^3+^ (1.16 Å) is larger than that of Ce^4+^ (0.97 Å), both in an eightfold coordination, the incorporation of La into the Ce site of CGO could explain the observed increase of the cell volume.^37^ It has to be also mentioned that minor cation interdiffusion between LCM and CGO is not expected to have detrimental effects on the electrochemical properties. For instance, it is reported that Ce doping in LaCrO_3−δ_ improves the performance due to the high catalytic activity of Ce^4+^/Ce^3+^ species for fuel oxidation.^38^ It is also worth noting that the phase quantification, determined by Rietveld analysis, is similar to the nominal one, further confirming the composition of the nanocomposite electrodes (Table 1).

The crystallite sizes are estimated by Scherrer′s equation, taking into consideration the instrumental peak broadening with a LaB_6_ standard powder sample. This takes values of ∼10 nm for CGO and 30 nm for LCM at 800 °C in the nanocomposite electrode (Table 1), and more interestingly, the grain growth is clearly inhibited at high sintering temperatures with a value lower than 40 nm at 1000 °C. In contrast, 100LCM has a larger crystallite size of 59 nm at 800 °C and grows up to 200 nm at 1000 °C.

The phase stability of nanocomposite electrodes was investigated at a high annealing temperature and under a reducing atmosphere. No additional diffraction peaks, attributed to secondary phases, were observed after treating the nanocomposite layers at 1000 °C for 5 h, in accordance with previous findings for related materials (Figure 1c and Table 1).^39^ Moreover, the nanocomposite electrodes were redox stable in H_2_ with no significant differences in the phase content and crystallite size (Table 1 and Figure S1). Nevertheless, the unit cell of LCM slightly expands after reduction in H_2_ due to partial reduction of the B-site cations to lower oxidation states with the consequent increase of the ionic radii (Table 1 and Figure S1b).

Similar results are observed for the nanocomposite electrodes deposited on polycrystalline YSZ and LSGM electrolytes. In this case, three crystalline phases are considered to fit the XRD patterns, i.e., LCM (s.g. *Pbnm*), CGO (s.g. *Fm*3̅*m*), and YSZ (cubic, s.g. *Fm*3̅*m*) or LSGM (orthorhombic, s.g. *Imma*) substrates. Representative Rietveld plots for 50LCM are displayed in Figure S1c,d and the structural parameters are summarized in Table 1.

### Microstructure

**Figure 2:**
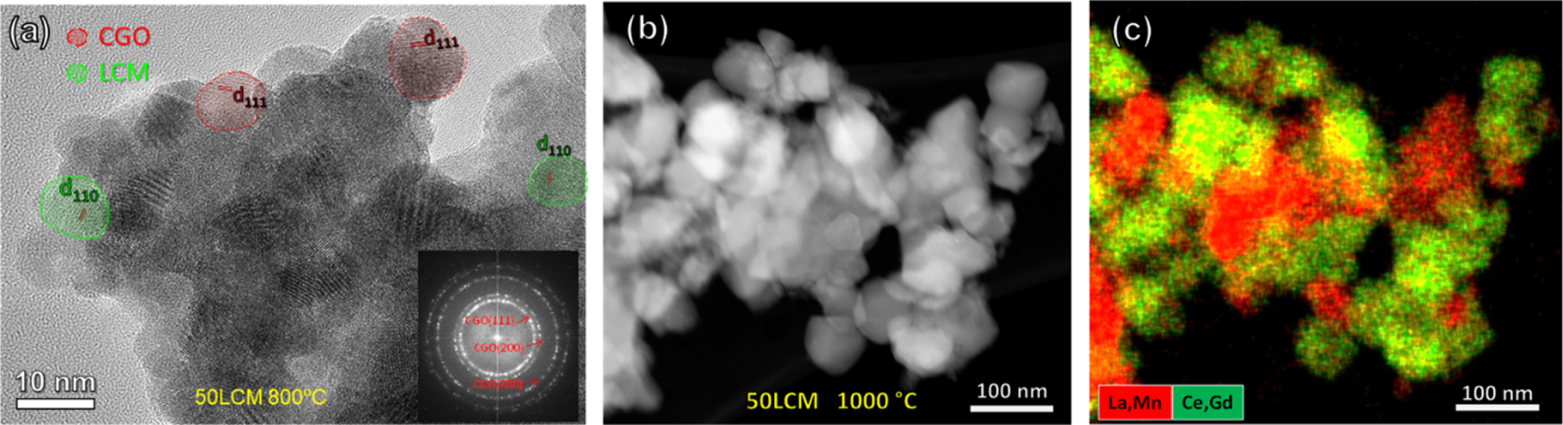
(a) HRTEM image of 50LCM calcined at 800 °C for 1 h. The inset shows the electron diffraction pattern, where the main reflections are assigned to a cubic fluorite. (b) HAADF-STEM image of 50LCM calcined at 1000 °C and the corresponding (c) EDX elemental mapping distribution.

Figure 2a shows the high-resolution transmission electron microscopy (HRTEM) image of 50LCM calcined at 800 °C, revealing that the nanocomposite is formed by particle aggregates ∼10 nm in diameter, which is consistent with the XRD results (Table 1). The measured interplanar distances *d_hkl_* correspond to a mixture of crystals with cubic fluorite and orthorhombic perovskite-type structures, without visible formation of amorphous domains. The most intense reflections in the electron diffraction patterns are assigned to a cubic fluorite, while the weak reflections of LCM with a lower crystal symmetry are not clearly discernible (inset Figure 2a). Interestingly, the grain size remained below 50 nm after annealing at 1000 °C for 5 h, confirming the high thermal stability of the nanocomposite electrodes (Figure 2b). HAADF-STEM and energy-dispersive X-ray (EDX) analysis also revealed that CGO and LCM particles are homogeneously distributed, ensuring good percolation between both the phases (Figure 2c).

**Figure 3:**
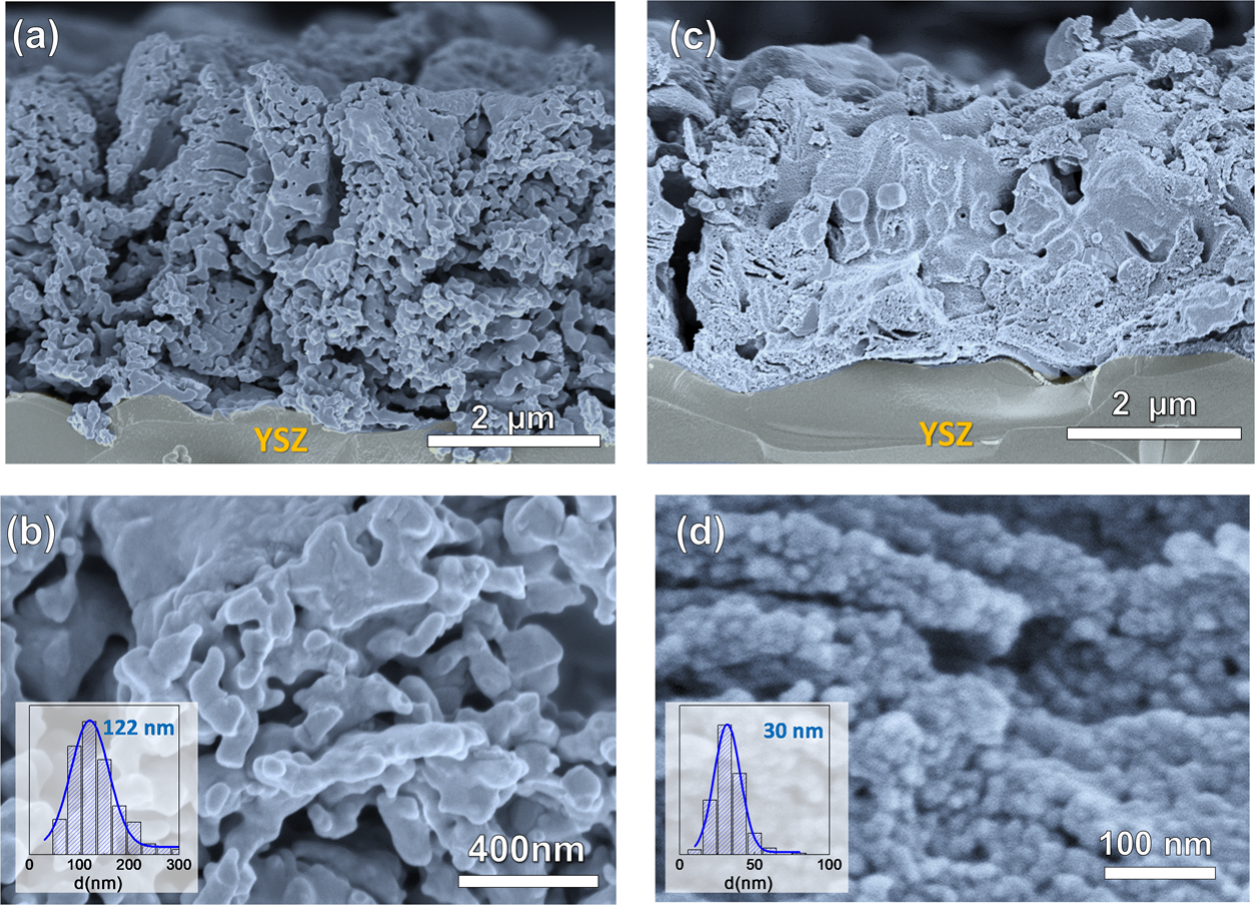
SEM image of (a, b) 100LCM and (c, d) 50LCM deposited on YSZ at different magnifications. The insets show the grain size distribution.

Cross-sectional SEM images of the cell show a porous morphology for both 100LCM and 50LCM (Figure 3a,b). The thickness of both the layers is similar, ∼3 μm. The main difference between both the electrodes is the average grain size with a value of 122 nm for 100LCM and 30 nm for 50LCM after long-term annealing at 800 °C (Figure 3c,d). Such a behavior is explained by the highly dispersed particles of two immiscible phases that limits the cation mobility during the sintering process, leading to nanosized grains.^25,40^ In comparison, the screen-printed 50LCM_SP electrode shows a much higher grain size about 500 nm due to the higher preparation temperature (Figure S2).

### Electrochemical Characterization

**Figure 4:**
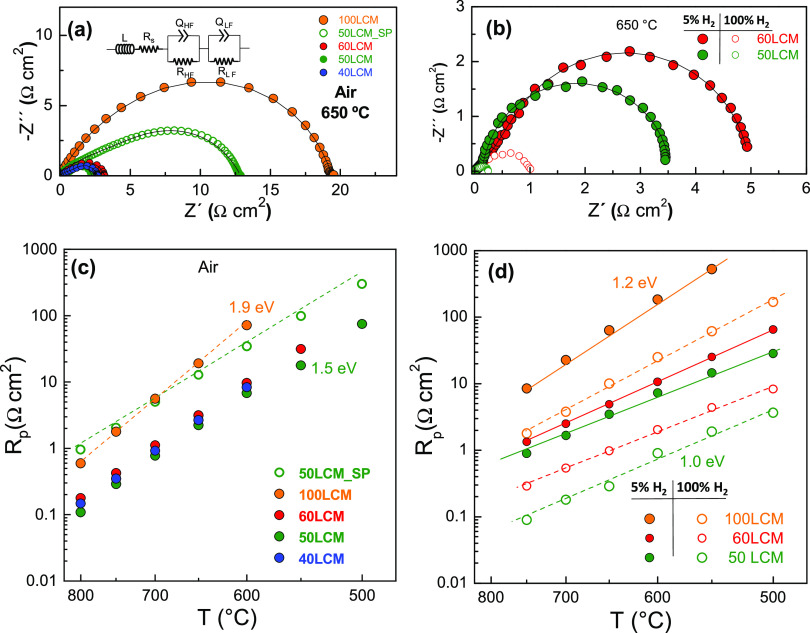
Impedance spectra of the different electrodes on the YSZ electrolyte at 650 °C in (a) air and (b) H_2_-containing atmosphere. Variation of the polarization resistance in (c) air and (d) H_2_. The equivalent circuit used to fit the impedance spectra is included in the inset of panel (a). Notice that the electrolyte resistance *R*_s_ was subtracted for better comparison of the electrode response.

Impedance spectra of the different nanocomposite electrodes in symmetrical cell configuration in air and H_2_ atmosphere at the open-circuit voltage are shown in Figure 4a,b, respectively. A screen-printed 50 wt % LCM-CGO composite (50LCM_SP), prepared by physically mixing powders, was included for comparison purposes.

To identify the electrochemical processes, the impedance spectra were deconvoluted by the DRT method.^41,42^ Two contributions denoted as HF and LF at high and low frequencies, respectively, were discernible in both oxidizing and reducing atmospheres for all electrode compositions (Figure S3). According to the DRT results, the spectra were adequately fitted using an equivalent circuit consisting of two RQ elements in series to simulate the high and low frequencies, HF and LF contributions, respectively, to the electrode polarization. In addition, a series resistance *R*_s_ and an inductance *L* are also included to take into account the ohmic contribution of the electrolyte and the inductive effects of the electrical connections, respectively (inset Figure 4a). The following parameters were obtained for each electrode contribution: the resistance *R*_*i*_, the pseudocapacitance *Q*_*i*_, and the exponential parameter *n*_*i*_. These parameters are related to the real capacitance *C*_*i*_ by the following relation^43^
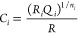


In oxidizing conditions, the screen-printed 50LCM_SP has capacitance values of about 0.01 and 0.1 mF cm^–2^ for the HF and LF responses, respectively, which are typical of a double layer capacitance, suggesting that these electrochemical processes are limited at the region between the electrode and the electrolyte interface (Figure S4a,b).^44^ In the case of the nanocomposite electrodes, the TPB length is significantly increased and higher capacitances are obtained, about 2 and 20 mF cm^–2^ for the HF and LF processes, respectively, regardless of the electrode composition of the nanocomposite. Regarding the resistance of the HF and LF responses, it is important to highlight that both contributions decrease significantly for the nanocomposite electrodes although 50LCM composition exhibits the lowest values (Figure S4c,d). In addition, the activation energies are similar with values of ∼1.4 and 1.7 eV for HF and LF processes, respectively.

In a 5% H_2_–Ar atmosphere, the LF response is the dominant contribution to the total polarization resistance. This process has a large capacitance of 0.2 F cm^–2^ and its resistance decreases abruptly in pure H_2_ from 1.67 to 0.11 Ω cm^2^ with a hydrogen partial partial dependence of (pH_2_)^0.92^, suggesting that it is possibly attributed to gas diffusion and concentration limitations (Figure S5).^42,45^ In contrast, the HF response with a capacitance of 15 mF cm^–2^ is less dependent on the H_2_ concentration and is associated with charge transfer processes at the electrode/electrolyte interface.

Electrode polarization studies were also performed on the LSGM electrolyte and the results were similar to those observed for the YSZ electrolyte (Figure S6). The impedance spectra are comprised of two processes (HF and LF) in both air and hydrogen atmospheres with capacitances and frequency values similar to those observed for the YSZ electrolyte. Thus, the same electrochemical processes seem to be involved in both LSGM and YSZ electrolytes. However, the total polarization resistance decreases slightly due to the higher ionic conductivity of LSGM compared to YSZ.

The total polarization resistance, *R*_p_ = *R*_HF_ + *R*_LF_, in air shows a pronounced decrease for the nanocomposite electrodes compared to the pure 100LCM with values at 750 °C of 1.78, 0.42, 0.35, and 0.29 Ω cm^2^ for 100LCM, 60LCM, 40LCM, and 50LCM, respectively (Figure 4c). It is also remarkable to mention that these values are almost one order of magnitude inferior to those obtained for the screen-printed 50LCM_SP composite electrode (∼2.05 Ω cm^2^). In addition, the activation energy of the polarization resistance is lower for the nanocomposite electrodes with values of 1.9 and 1.5 eV for 100LCM and 50LCM, respectively, implying that the oxygen reduction reaction kinetics is improved upon CGO addition.

In a reducing atmosphere, important differences are observed depending on the hydrogen concentration and CGO content in the nanocomposite electrodes (Figure 4d). First, the polarization resistance decreases in one order of magnitude when pure H_2_ is used instead of diluted hydrogen (5% H_2_–Ar), which is attributed to a drastic reduction of the LF contribution due to surface electrode processes. The optimal composition with the lowest polarization resistance is found for 50LCM, i.e., 0.09 Ω cm^2^ at 750 °C compared to 0.29 and 1.8 Ω cm^2^ for 60LCM and 100LCM, respectively. It is also worth noting that these values are lower than those reported for the most efficient symmetrical electrodes (Table 2), such as La_0.75_Sr_0.25_Cr_0.5_Mn_0.5_O_3−δ_ (0.30 Ω cm^2^ in H_2_ at 900 °C)^10^ and Sr_2_Fe_1.5_Mo_0.5_O_3−δ_ (0.45 Ω cm^2^ in H_2_ at 800 °C),^46^ and related anode materials with exsolved metal particles, i.e., La_0.75_Sr_0.25_Cr_0.5_Mn_0.3_Ni_0.2_O_3−δ_ (1.1 Ω cm^2^ at 800 °C in 5% H_2_)^47^ and Sm_0.8_Sr_0.2_Fe_0.8_Ti_0.15_Ru_0.05_O_3−δ_ (0.2 Ω cm^2^ at 800 °C in 5% H_2_).^48^ In addition, the activation energy values vary between 1.0 eV for 50LCM and 1.2 eV for 100LCM, and as expected, they are lower than those obtained in air atmosphere.

**Table 2:** Electrochemical Properties of Several LaCrO_3_-Based and Other Symmetrical Electrodes (SSOFCs) and LaCrO_3_ Anodes^a^

electrode	*R*_p_^air^ (Ω cm^2^)	*R*_p2_^H^ (Ω cm^2^)	*P* (mW cm^−2^)	electrolyte (thickness)	ref
100_LCM	1.78	1.80			this work
50_LCM SP	2.05	1.9			this work
50_LCM nanocomposite	0.29	0.09	571^800 °C^	LSGM (300 μm)	this work
			420^750 °C^		
La_0.98_Cr_0.75_Mn_0.25_O_3−δ_-CGO	0.14	0.32	270	LSGM (300 μm)	(30)
La_0.75_Sr_0.25_Cr_0.5_Mn_0.5_O_3−δ_	0.35^900 °C^ (wet O_2_)	0.30^900 °C^	300^900 °C^	YSZ (200 μm)	(10)
La_0.75_Sr_0.125_Ce_0.125_Cr_0.5_Mn_0.5_O_3−δ_			42^800 °C^ (3% H_2_)	YSZ (370 μm)	(49)
La_0.65_Bi_0.1_Sr_0.25_Cr_0.5_Fe_0.5_O_3_-SDC	8	0.4	175	LSGM (300 μm)	(16)
Sr_2_Fe_1.5_Mo_0.5_O_6−δ_ (SFM)	0.65	0.45	500^800 °C^	LSGM (265 μm)	(46)
Sr_2_Fe_1.5_Mo_0.5_O_6−δ_-SDC	0.29	0.12	250^800 °C^	LSGM (1200 μm)	(50)
La_0.3_Sr_0.7_Fe_0.9_Ti_0.1_O_3−δ_	0.04	0.26	600	LSGM (300 μm)	(51)
La_0.6_Sr_0.4_Fe_0.95_Pd_0.05_O_3−δ_-GDC	0.91	0.08 (5% H_2_)	350	LSGM (300 μm)	(52)

LaCrO_3_-based anodes	cathode	*R*_p2_^H^ (Ω cm^2^)	*P* (mW cm^−2^)	electrolyte (thickness)	ref
La_0.3_Ca_0.6_Ce_0.1_CrO_3−δ_	LSCF	0.80^760 °C^	220	LSGM	(53)
La_0.8_Sr_0.2_Cr_0.5_Fe_0.5_O_3−δ_-LSGM	LaNi_0.6_Fe_0.4_O_3_	0.32	765	LSGM (300 μm)	(54)
La_0.6_Sr_0.4_Cr_0.6_Fe_0.4_O_3−δ_	LSCF-CGO	0.30^800 °C^	325	LSGM (400 μm)	(55)
La_0.65_Bi_0.1_Sr_0.25_Cr_0.5_Fe_0.5_O_3_-SDC	LSCF-SDC	0.4	380	LSGM (300 μm)	(16)
La_0.8_Sr_0.2_Cr_0.9_Pd_0.1_O_3−δ_-CGO	LSCF		303^800 °C^	LSGM (400 μm)	(56)
La_0.8_Sr_0.2_Cr_0.82_Ru_0.18_O_3−δ_-CGO	LSCF-CGO		530^800 °C^	LSGM (400 μm)	(57)

a Polarization resistance (*R*_p_) values in air and pure H_2_ are given at 750 °C. Temperature is included when data are not available at 750 °C.

**Figure 5:**
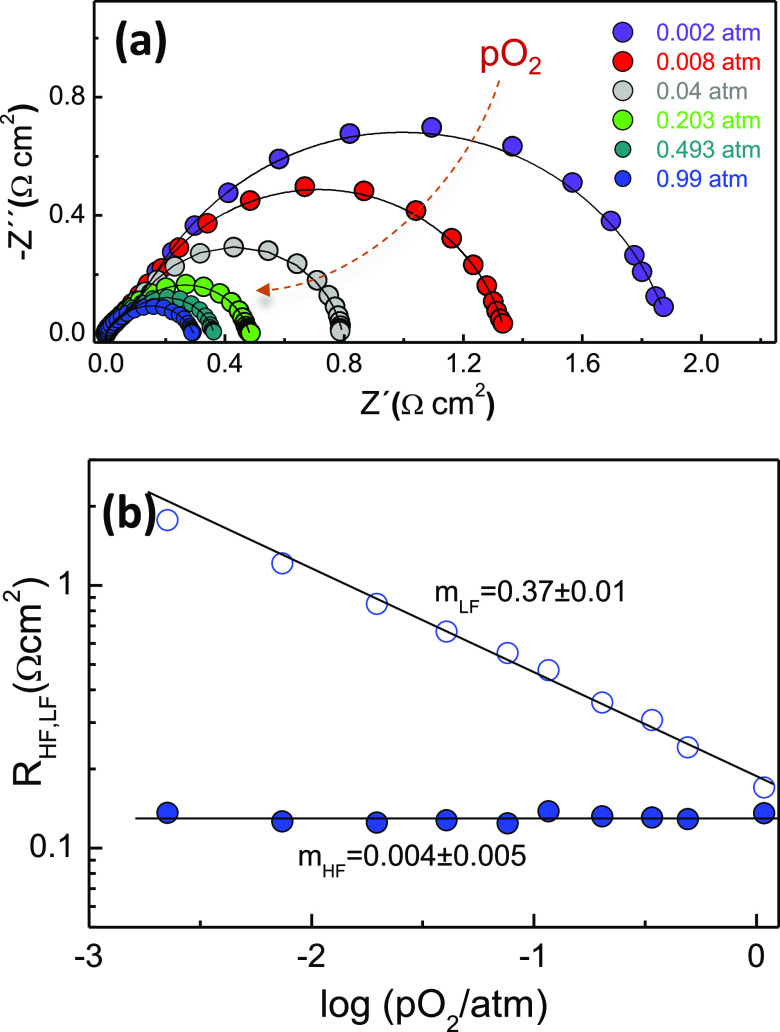
(a) Impedance spectra of 50LCM on the YSZ electrolyte at 700 °C at different oxygen partial pressures, pO_2_. (b) Dependence of the HF and LF resistances as a function of pO_2_.

Since 50LCM exhibits improved performance as both fuel and air electrodes, this composition was further characterized by other electrochemical techniques to obtain further insights on the nature of the electrochemical processes. The impedance spectra were acquired at different oxygen partial pressures (pO_2_) to identify the different processes involved in the oxygen reduction reaction (Figure 5a). The relationship between the resistance of different electrochemical processes and pO_2_ is described using the following expression: *R*_HF,LF_ ∼ (pO_2_)^−*m*^, where *m* provides information about the type of species involved in each process.^58^

The LF contribution, which is the dominant step, with *m* close to 3/8 is associated with dissociation and adsorption of oxygen, the formation of adsorbed oxygen ions, or the surface diffusion of oxygen atoms: O_ad_ + e^–^ → O_ad_^–^.^59^ In contrast, the HF response is insensitive to pO_2_, implying that atomic and molecular oxygen are not involved in this process, and therefore, this is associated with oxygen ion incorporation from the TPB to the electrolyte: O_TPB_^2–^ + *V*_Ö_ → O_o_^x^.^20,60−62^

**Figure 6:**
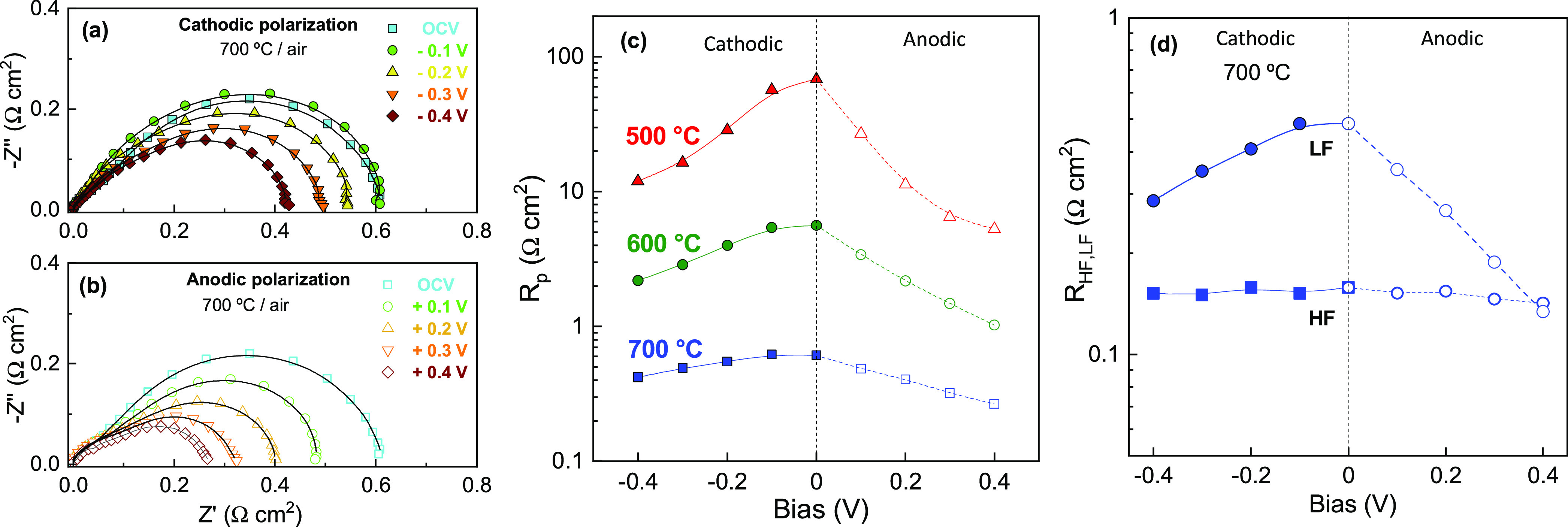
Impedance spectra of 50LCM acquired in a three-probe configuration at different dc bias under: (a) cathodic and (b) anodic polarization at 700 °C in air atmosphere. (c) Total electrode polarization resistance as a function of the dc bias at different temperatures. (d) Variation of HF and LF contributions to the electrode polarization resistance at 700 °C.

The influence of the dc current bias on the electrode polarization was also investigated by a three-probe electrode configuration. Figure 6a,b shows the impedance spectra in air acquired under cathodic and anodic polarizations, respectively. It is worth noting that the values of polarization resistance are similar to those obtained in a two symmetrical cell configuration. The polarization resistance under cathodic polarization at 700 °C decreases from 0.6 Ω cm^2^ at OCV to 0.41 Ω cm^2^ at −0.4 V, which is attributable to the formation of oxygen vacancies in the lattice after the application of the dc bias.^34^ In particular, the current density plays an important role in accumulating or removing oxygen vacancies at the reaction sites, as well as increases the diffusivity of vacancies. For instance, a dc bias in La_0.8_Sr_0.2_MnO_3−δ_ partially reduces Mn^3+^ to Mn^2+^ with the consequent formation of oxygen vacancies, enhancing the electrochemical properties.^63^ The effect of the dc bias on the electrode response is more important at the low temperature range, varying from 68 to 11 Ω cm^2^ at 500 °C, under cathodic polarization. When the dc polarization is reversed to the anodic mode, the *R*_p_ is further reduced with a value at 0.4 V of 5.3 Ω cm^2^ at 500 °C and 0.26 Ω cm^2^ at 700 °C (Figure 6c). The better results in anode polarization are attributed to an increase of the oxygen partial pressure at the oxygen electrode, enhancing the electron–hole conductivity of both CGO and LCM materials. The spectra were analyzed by equivalent circuits to study the evolution of *R*_HF_ and *R*_LF_ resistances under polarization (Figure 6d). The *R*_HF_ process remains almost invariant with the applied dc bias due to the high mixed ionic–electronic conductivity of the nanocomposite electrode and fast charge transfer at the electrode/electrolyte interface.^34,61^ Conversely, the *R*_LF_ process, associated with electrode surface steps, lowered with increasing dc bias, a trend similar to that observed in the electrode polarization dependence on pO_2_, and also reported for other commonly used air electrodes, such as La_2_NiO_4+δ_, SrCo_0.9_Mo_0.1_O_3−δ_, and SrCo_0.9_Ta_0.1_O_3−δ_.^34,64,65^

The measurements performed in wet 100% H_2_ reveal a significant reduction of the polarization resistance under cathodic polarization from 0.29 to 0.15 Ω cm^2^ at 700 °C (Figure S7). However, when an anodic polarization is applied, the polarization resistance increases up to 0.55 Ω cm^2^ at +0.4 V. The HF process, assigned to the charge transfer process, remains constant under dc bias in both anodic and cathodic polarizations. In contrast, the LF process decreases under cathodic polarization and increases under anodic polarization. A similar behavior has been observed for La_0.3_Sr_0.7_Fe_0.7_Cr_0.3_O_3−δ_ and Sr_1.8_Ba_0.2_Fe_1.5_Mo_0.5_O_6−δ_ under anodic polarization; however, additional studies at different pH_2_ and pH_2_O values are needed to better understand and identify the different processes involved in the hydrogen oxidation reaction (HOR) and hydrogen evolution reaction (HER) in these nanocomposite electrodes.^66,67^

### Single Cell Tests

**Figure 7:**
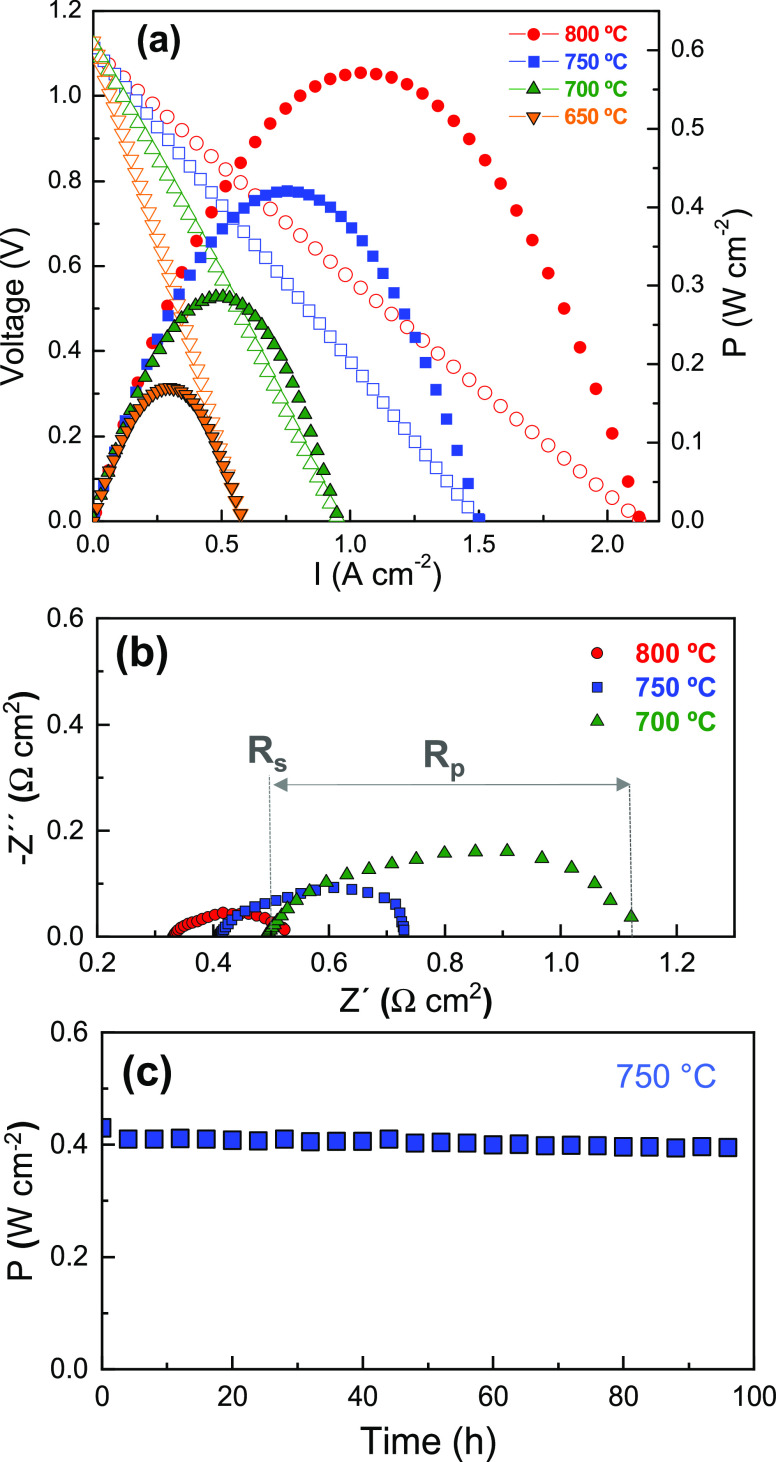
(a) *I*–*V* and power density curves of the symmetrical cell with the 50LCM electrode over 300-μm-thick LSGM electrolyte at different temperatures using wet H_2_ as fuel. (b) Impedance spectra and (c) maximum power density of the cell over time at an applied constant voltage of 0.55 V at 750 °C.

The performance of the nanocomposite electrodes in real SOFC operation conditions was tested in an electrolyte-supported cell in symmetrical configuration: 50LCM/LSGM/50LCM. Current–voltage and power density curves, using air as oxidant and humidified H_2_ as fuel, are displayed in Figure 7a. The open-circuit voltage is comparable to the theoretical Nernst potential (OCV = 1.1 V), confirming a good sealing of the cell. On the other hand, the ohmic resistance *R*_s_ increases with decreasing temperature from 0.32 to 0.50 Ω cm^2^ at 800 and 700 °C, respectively, which are in good agreement with the estimated values for a 300-μm-thick LSGM electrolyte (Figure 7b).^68^ In addition, the values of polarization resistance, i.e., *R*_p_ = 0.18 Ω cm^2^ at 800 °C, are lower than the corresponding ohmic resistance, suggesting that the fuel cell output could be improved by reducing the electrolyte thickness.

The symmetrical cell generates maximum power densities of 570, 420, and 286 mW cm^–2^ at 800, 750, and 700 °C, respectively. It should be highlighted that the performance of this cell is one of the best reported to date for a LaCrO_3_-based symmetrical electrode, e.g., 300 mW cm^–2^ at 900 °C for La_0.75_Sr_0.25_Cr_0.5_Mn_0.5_O_3−δ_ over a 200 μm YSZ-electrolyte-supported cell and 175 mW cm^–2^ at 800 °C for La_0.65_Bi_0.1_Sr_0.25_Cr_0.5_Fe_0.5_O_3_-SDC over a 300-μm-thick LSGM electrolyte (Table 2). In addition, the electrochemical performance of 50LCM is also superior to that of LaCrO_3_-based anodes with exsolved noble metal particles, such as La_0.8_Sr_0.2_Cr_0.9_Pd_0.1_O_3−δ_-CGO and La_0.8_Sr_0.2_Cr_0.82_Ru_0.18_O_3−δ_-CGO (Table 2).

Finally, a short stability test was carried out at 750 °C for 100 h and the peak power density of the cell, obtained by applying a constant voltage of 0.55 V over time, remained almost constant (Figure 7c). Moreover, no appreciable microstructural changes of the electrodes were observed after the electrochemical test, confirming the stability of the LCM-CGO nanocomposite at intermediate temperatures (Figure S8).

In summary, better electrochemical properties of LCM-CGO nanocomposites are associated with several factors: (i) the low preparation temperature, which minimizes the chemical reactivity at the electrolyte/electrode interface. (ii) The self-assembled nanocomposite electrodes exhibit a strong interphase interaction, reducing the thermal expansion and improving the physical compatibility and adherence to the electrolyte. (iii) The low particle size of the nanocomposite electrodes significantly increases the TPB length, and consequently, the efficiency. (iv) The synergetic effect between LCM and CGO with high electronic and ionic conductivities, respectively. (v) Ceria is also known to release more lattice oxygen when decreasing the particle size, and it exhibits high mixed ionic–electronic conductivity under reducing conditions due to Ce^4+^ to Ce^3+^ reduction.^69^ (vi) Moreover, these nanocomposite electrodes are alkaline-earth-free, making them less susceptible to carbonation and surface phase segregation, which usually deteriorate the electrochemical properties after long-term operation.^70^ (vii) Moreover, recently, nanoscale La_0.9_Sr_0.1_CrO_3−δ_ has been reported to exhibit an enhancement of the oxygen ion diffusivity and surface exchange coefficients compared to the bulk.^71^ Thus, the combination of all of these properties provides suitable nanocomposites to operate simultaneously as both air and fuel electrodes and could be the key for the development of new symmetrical electrodes for SOCs.

## Conclusions

Nanocomposite electrodes with a nominal composition of *x*·La_0.98_Cr_0.75_Mn_0.25_O_3−δ_–Ce_0.9_Gd_0.1_O_1.95_ (*x* = 0–60 wt %) were prepared for the first time in a single cosynthesis step by spray-pyrolysis deposition, reducing the fabrication time and costs compared to a traditional and physically mixed composite electrode deposited by the screen-printing method. The structural and microstructural evolution with the annealing temperature confirmed that CGO and LCM phases are practically immiscible in the temperature range studied up to 1000 °C. Moreover, the nanocomposite electrodes presented high redox stability, which makes them suitable for both air and fuel electrodes.

The strong interphase interaction between the CGO and LCM nanoparticles limited the grain growth rate and improved the thermal compatibility with the electrolyte. Interestingly, the nanometric particle size is retained at high annealing temperatures with values as low as 10 and 50 nm at 800 and 1000 °C, respectively. The higher nanoscale contact between the CGO and LCM particles led to larger concentration of catalytic active centers compared to a screen-printing electrode, and consequently, the polarization resistance was greatly reduced from 2.05 to 0.29 Ω cm^2^ in air at 750 °C.

The 50LCM symmetrical electrode over a LSGM-supported cell showed a remarkable maximum power density of 570 mW cm^–2^ at 800 °C, higher than that reported previously for a LaCrO_3_-based electrode. Thus, the preparation of nanocomposites by a cosynthesis method is a promising strategy to combine the electrochemical properties of multiple phases to design new highly efficient and durable symmetrical electrodes for application in fuel cell and solid electrolyzer cells.

## Supporting Information Available

Rietveld plots; SEM image of screen-printed 50LCM; DRT spectra; capacitance and polarization resistance in air; resistance contributions in hydrogen; impedance, DRT spectra, SEM, and polarization resistance on the LSGM electrolyte; impedance spectra, total polarization resistance, and different contributions (PDF)
