# Supplementary material for: LaCrO3–CeO2-Based Nanocomposite Electrodes for Efficient Symmetrical Solid Oxide Fuel Cells
Source: ACS Appl Energy Mater. 2022 Apr 5;5(4):4536–46. doi: 10.1021/acsaem.1c04116 (PMC9513820; doi:10.1021/acsaem.1c04116)
Supplement: Supplementary file 1 — ae1c04116_si_001.pdf [file ae1c04116_si_001.pdf]

## Supporting information

# $LaCrO_3$-$CeO_2$ based nanocomposite electrodes for efficient symmetrical Solid Oxide Fuel Cells

*Javier Zamudio-García*[†], *José M. Porras-Vázquez*[†], *Enrique R. Losilla*[†], *David Marrero-López*[§,*]

[†]Universidad de Málaga, Departamento de Química Inorgánica, Campus de Teatinos s/n, 29071-Málaga, Spain.

[§]Universidad de Málaga, Departamento de Física Aplicada I, Campus de Teatinos s/n, 29071-Málaga, Spain.

* Corresponding author: marrero@uma.es

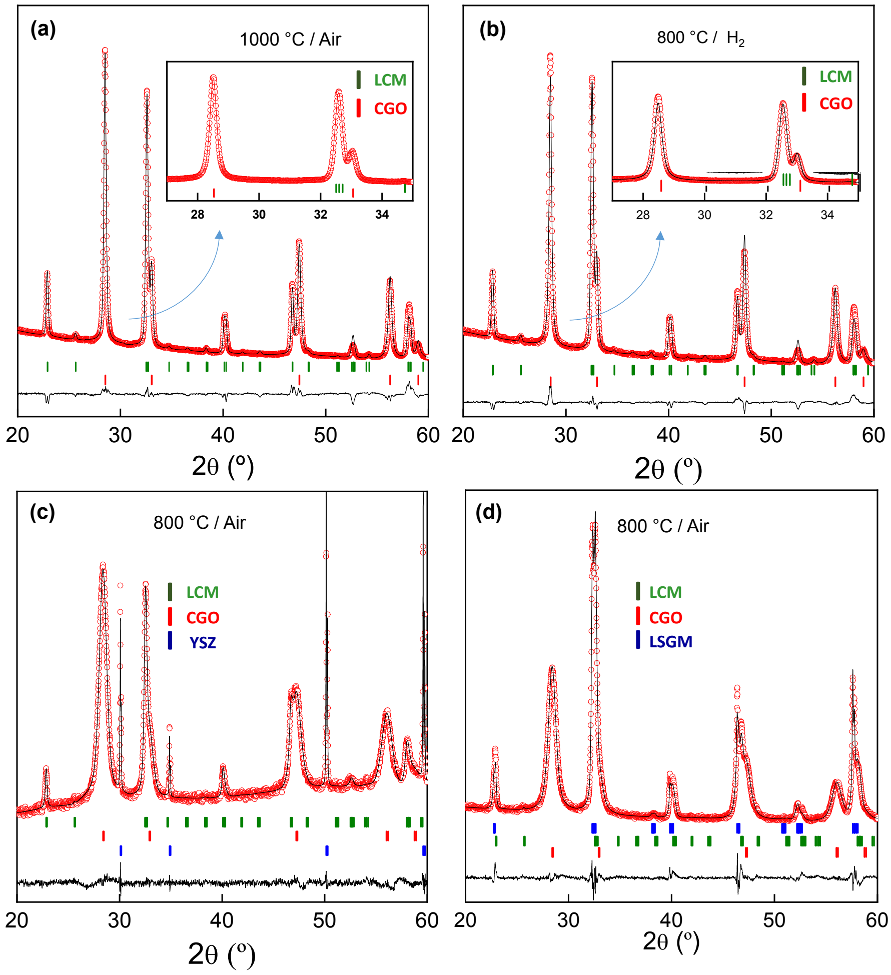


**Figure S1.** Rietveld plots of 50LCM deposited on quartz substrate after annealing at (a) 1000 ºC in air and (b) 5%$H_2$-Ar at 800 ºC for 5 h. Rietveld plots of 50LCM deposited on (c) YSZ and (d) LSGM pellets after calcining at 800 ºC in air.

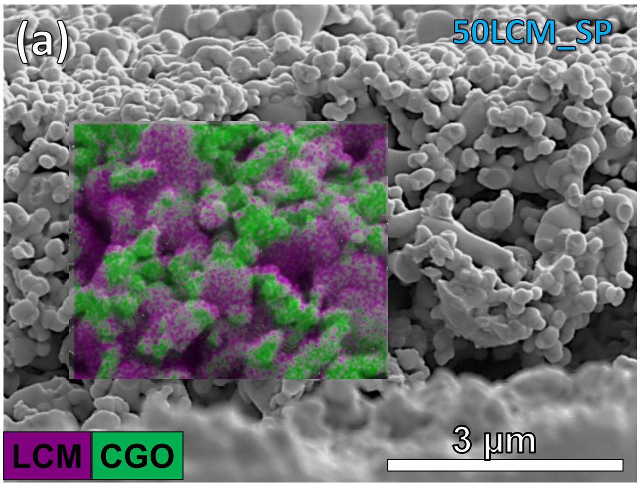


**Figure S2**. SEM image of the 50LCM obtained by screen-printing deposition.

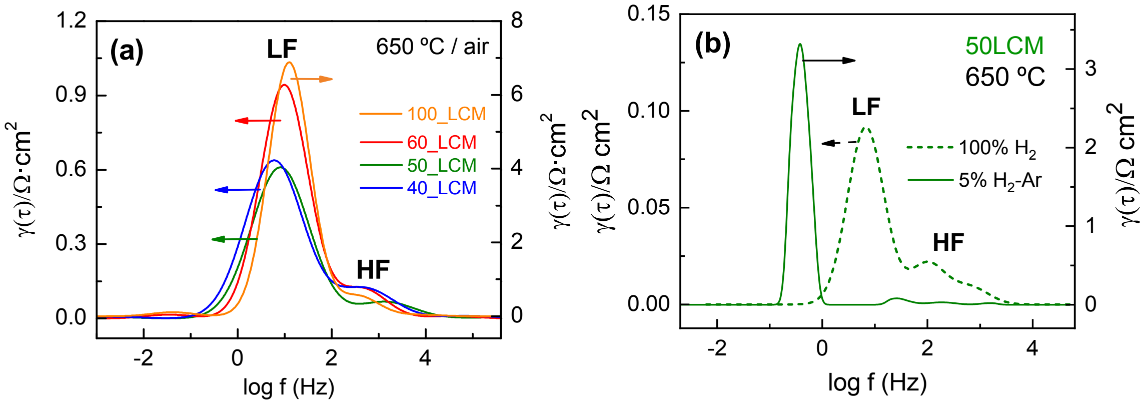


**Figure S3**. DRT spectra of (a) the different LCM-CGO electrodes at 650 ºC in air and (b) 5% $H_2$-Ar and 100% $H_2$ for 50LCM.

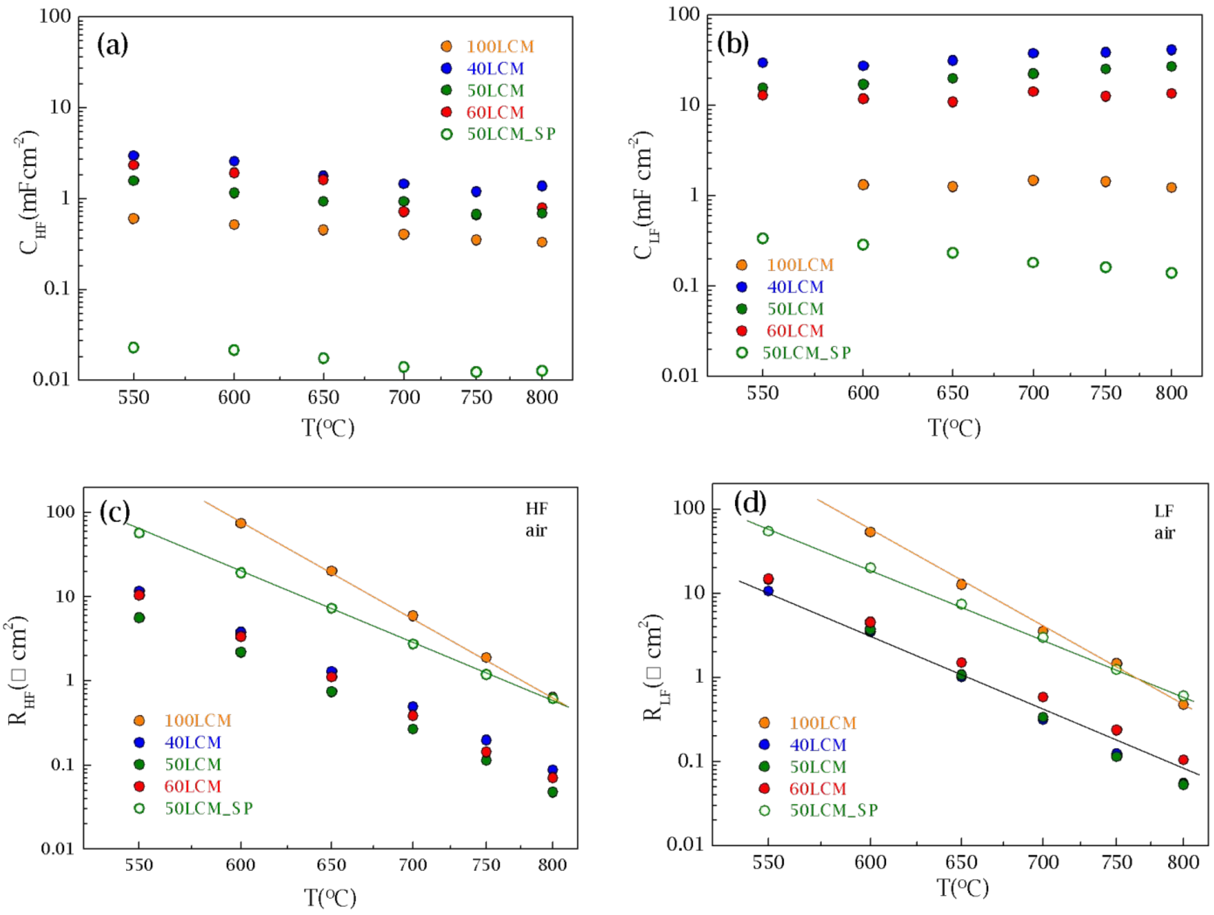


**Figure S4.** Variation of capacitance with temperature of the (a) HF and (b) LF contribution of the polarization in air. Temperature dependence of the (c) HF and (d) LF contribution to the polarization in air.

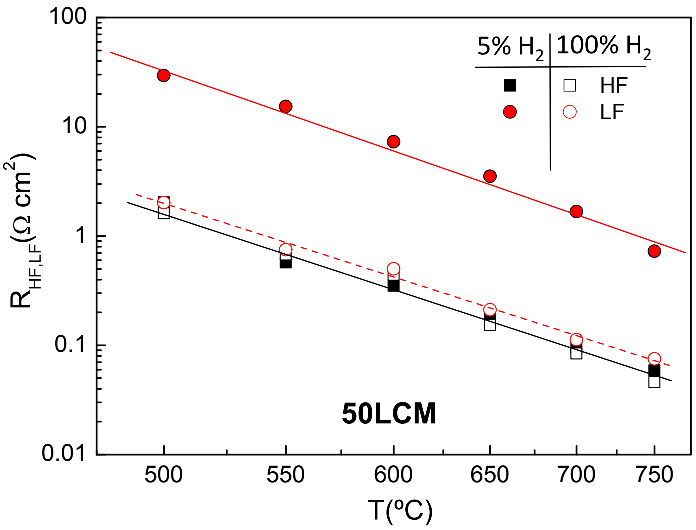


**Figure S5.** Temperature dependence of the HF and LF contribution to the electrode resistance polarization in 5% $H_2$-Ar and wet 100% $H_2$.

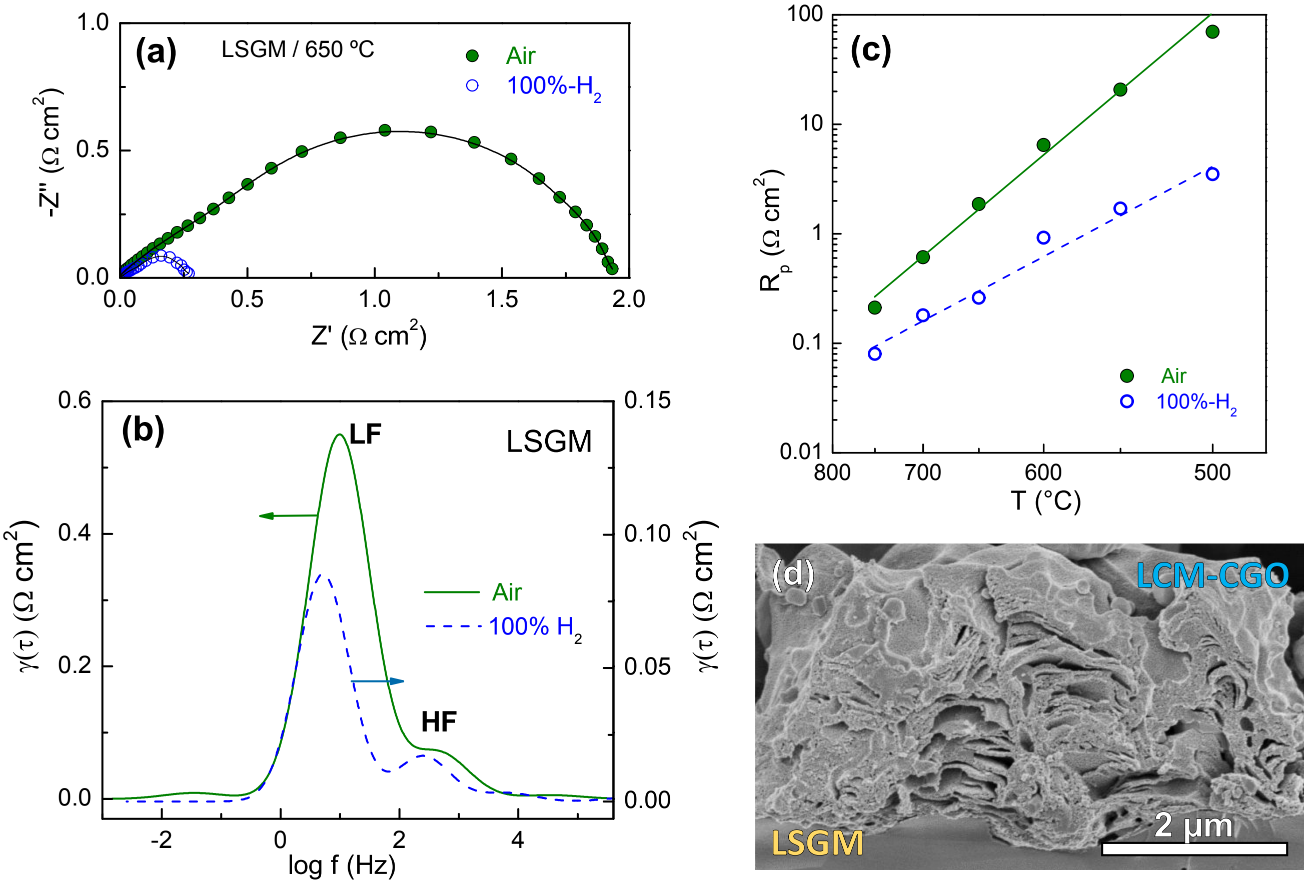


**Figure. S6.** (a) Impedance spectra at 650 ºC, (b) DRT spectra at 650 ºC, (c) total polarization resistance of 50LCM deposited on LSGM electrolyte in both air and wet $H_2$ and (d) SEM image of 50LCM electrode.

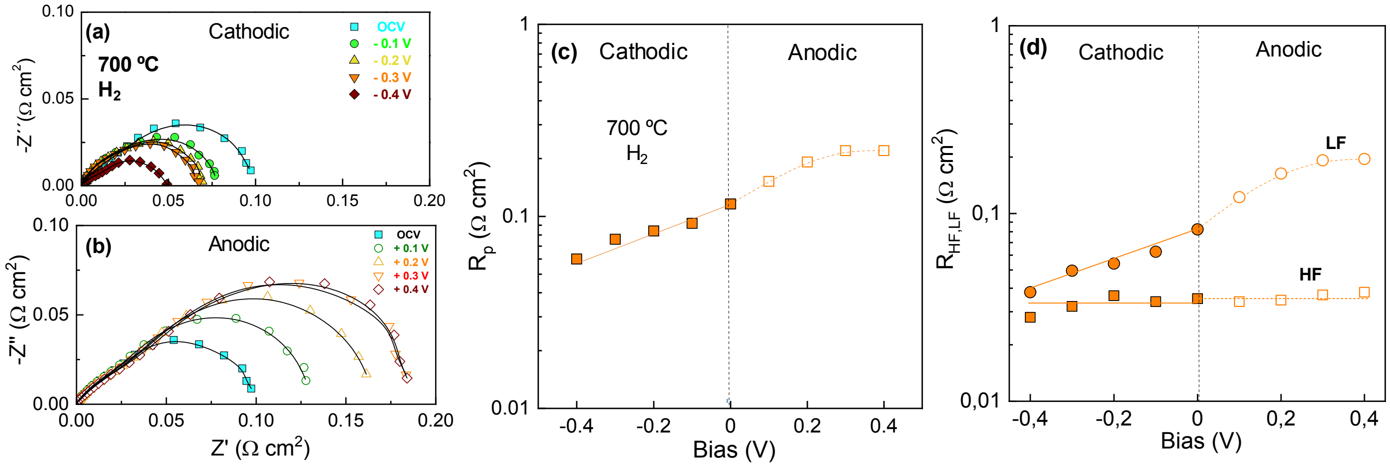


**Figure S7.** Impedance spectra of 50LCM acquired in pure $H_2$ in 3-probe configuration at different dc-bias under (a) cathodic and (b) anodic polarization at 700 ºC. (c) Overall electrode polarization resistance as function of the dc-bias at different temperatures and (d) Variation of the HF and LF contributions to the electrode polarization resistance at 700 ºC in wet $H_2$.

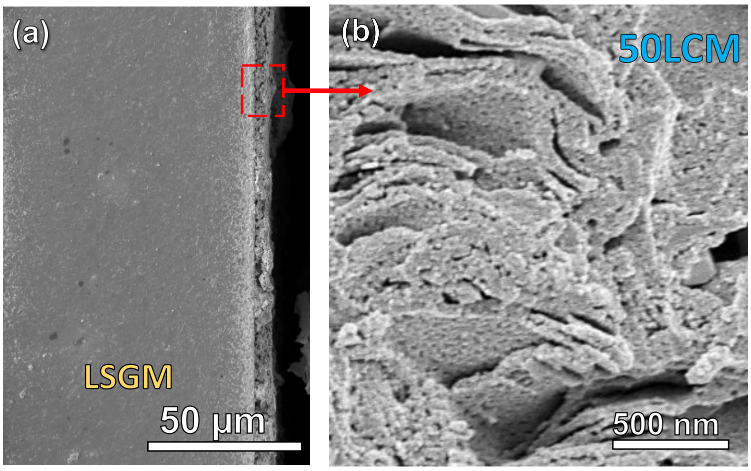


**Figure S8** a) Cross-sectional SEM image 50LCM / LSGM / 50LCM and (b) electrode microstructure after the electrochemical test.
